# Association between the clinical features of and types of temporomandibular joint ankylosis based on a modified classification system

**DOI:** 10.1038/s41598-019-46519-8

**Published:** 2019-07-19

**Authors:** Long Xia, Jingang An, Yang He, E. Xiao, Shuo Chen, Yingbin Yan, Yi Zhang

**Affiliations:** 1 Department of Oral and Maxillofacial Surgery, Peking University School and Hospital of Stomatology, 22 Zhongguancun South Avenue, Beijing, 100081 P. R. China; 2 Department of Oral and Maxillofacial Surgery, Tianjin Stomatological Hospital, 75 Dagu Road, Heping District, Tianjin 300041 P. R. China

**Keywords:** Oral diseases, Trauma, Diagnosis

## Abstract

This study aimed to describe the clinical features of different types of traumatic temporomandibular joint (TMJ) ankylosis. Seventy-one patients with 102 ankylosed joints were retrospectively reviewed and categorized into four groups according to the grades of severity: type I, non-bony ankylosis of the joint with almost-normal joint space; type II, lateral bony ankylosis marked by a normal joint space that coexists with a radiolucent line; type III, complete bony ankylosis of the joint characterized by only a radiolucent line; and type IV, extensive bony ankylosis without any radiolucent line. The period of ankylosis, maximal mouth opening (MMO), rate of complications, and histopathological changes were compared among groups. Intergroup comparison showed significant differences in the clinical features of MMO and the incidence of complications (p < 0.05). Younger trauma patients tended to develop more severe types of ankylosis than older patients. Additionally, long post-trauma periods were related to the development of severe ankylosis. MMO was highly negatively correlated with the severity of ankylosis. Significant differences were noted among the four types of ankylosis. Younger trauma patients with long post-trauma periods tended to develop more severe TMJ ankylosis, experience more complications, and face more challenges in treatment than older patients.

## Introduction

Traumatic temporomandibular joint (TMJ) ankylosis refers to the fibrous or bony fusion between the condyle and fossa, which is often caused by condylar fracture. It usually develops before the age of 10 years^1^, but could develop at any age^2^. The main clinical features of TMJ ankylosis are progressive limitation of mouth opening, facial deformity, and obstructive sleep apnea syndrome (OSAS)^3–6^. Patients usually present with limitation of mouth opening and a maximum interincisal distance of 0–20 mm. This condition eventually causes aesthetic defects in the face, malocclusion, and facial malformation, particularly during childhood^4^.

Our previous study revealed a correlation between mouth opening and the Sawhney class in patients, suggesting that the extent of bony fusion area influences mouth opening^7^. However, a few patients with Sawhney class III or IV could open their mouth wider than those with Sawhney class II, which suggested that the extent of bony fusion area was not the only factor influencing mouth opening^8^. Moreover, we believe that previous classifications do not adequately address the clinical features of TMJ ankylosis and the disease-development process.

Based on findings from previous studies, a radiolucent zone in the ankylosis mass could explain the pathogenesis of TMJ ankylosis^9^, reflect the disease progression^10^, and be related to mouth opening^8^. We hypothesized that the radiolucent zone may represent the course of bone healing of two traumatic articular surfaces with interference from the mouth-opening movement. The characteristic images of computed tomography (CT) could be used to indicate the developmental stage of TMJ ankylosis and possibly help in identification of disease severity and treatment options. However, the aforementioned studies focused more on basic medical research and lacked convincing proof to describe the association with clinical presentation.

Therefore, this study aimed to describe the clinical features of different types of TMJ ankylosis as per our proposed classification and validate the correlations between the severity of clinical features and the type of disease.

## Results

### Types of TMJ ankylosis

**Figure 1:**
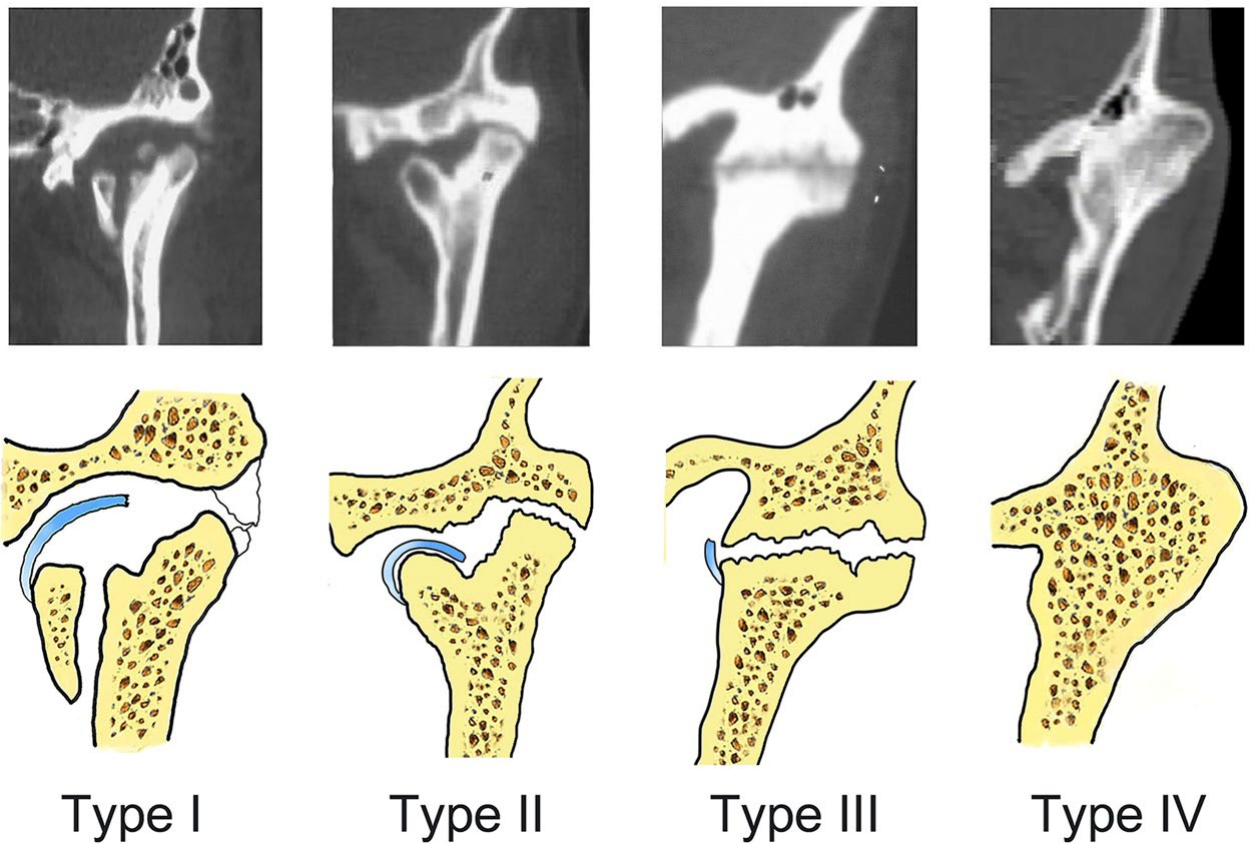
Computed tomography images and representations of the four types of temporomandibular joint ankylosis. Type I is nonbony ankylosis of the joint, with the fossa and condyle clearly seen but with scattered callus. Type II is lateral bony ankylosis of the joint, with bony fusion on the lateral side of the joint. The medially displaced condyle, residual disc, and fossa form a pseudarthrosis. Type III is complete bony ankylosis of the entire joint with a radiolucent line inside the fusion area but no recognizable condyle and fossa. Type IV is extensive bony ankylosis, with complete disappearance of the joint and with no radiolucent line.

**Figure 2:**
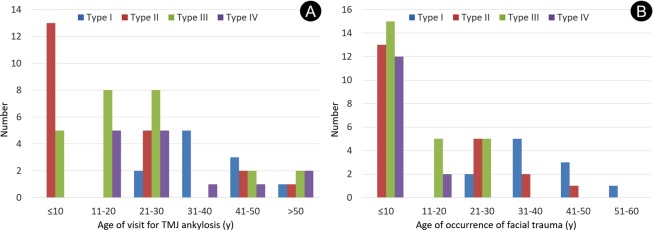
Age distributions of patients across the four types of temporomandibular joint ankylosis. (**A**) At the time of visit for diagnosis of TMJ ankylosis; (**B**) at the time of occurrence of facial trauma.

As per our method of classification of the 71 cases (Fig. 1), 11 patients had type I, 21 patients had type II, 25 patients had type III, and 14 patients had type IV ankylosis. The coverage rate was 100%, and the Kappa value was 0.922 (p < 0.05), indicating an extremely high interobserver agreement. The mean patient age was 24 (range 5–63) years, and the mean age at the occurrence of maxillofacial trauma was 16 (range 1–60) years (Fig. 2). Type IV ankylosis was observed in patients with facial trauma aged <20 years alone; similarly, type III ankylosis was observed in patients injured age <30 years alone. On the other hand, type I ankylosis was found in injured patients aged >20 years alone, whereas type II ankylosis was found in injured patients of all ages.

### Clinical characteristics

Of the 71 patients, 21 presented with mandibular asymmetry, 22 presented with mandibular retrusion, and 5 experienced OSAS. All patients presented with limited mouth opening; the maximum interincisal distance at presentation ranged 0–30 mm.

Table 1 summarizes results of clinical examinations as well as rates of complications in each group. χ^2^ and Fisher’s exact tests showed no statistical significance in the mandibular asymmetry among types II, III, and IV (p > 0.05). In contrast, significant differences were observed in mandibular retrusion and combined OSAS between type III and type IV patients (p < 0.05).

**Table 1:** Clinical examination findings and rates of complications as per the modified classification method.

Types	Patients	Gender(Male/Female)	Age of visit for TMJ ankylosis (y)	Age of occurrence of facial trauma (y)	MMO(mm)	Mandibular asymmetry(%)	Mandibular retrusion(%)	OSAS(%)
**I**	11	8/3	39.6 ± 10.4	39.2 ± 10.6	14.9 ± 8.2	0	0	0
**II**	21	10/11	17.3 ± 13.8	14.6 ± 12.4	11.6 ± 6.6	7 (33%)	2 (9%)	0
**III**	25	14/11	22.3 ± 13.5	11.1 ± 8.3	7.4 ± 4.9	9 (36%)	8 (32%)	1 (4%)
**IV**	14	10/4	29.2 ± 16	7 ± 2.9	1.9 ± 2.9^a^	5 (36%)^b^	12 (86%)^c^	4 (29%)^d^
**Total**	71	42/29				21	22	5

TMJ: temporomandibular joint; MMO: maximal mouth opening; OSAS: obstructive sleep apnea syndrome; ^a^p < 0.05, intergroup comparisons among types I, II, III, and IV for maximal mouth opening; ^b^p > 0.05, intergroup comparisons among types II, III, and IV for rate of mandibular asymmetry; ^c^p < 0.05, intergroup ^c^omparisons between types III and IV for rate of mandibular retrusion; ^d^p < 0.05, intergroup comparisons between types III and IV for rate of OSAS.

**Figure 3:**
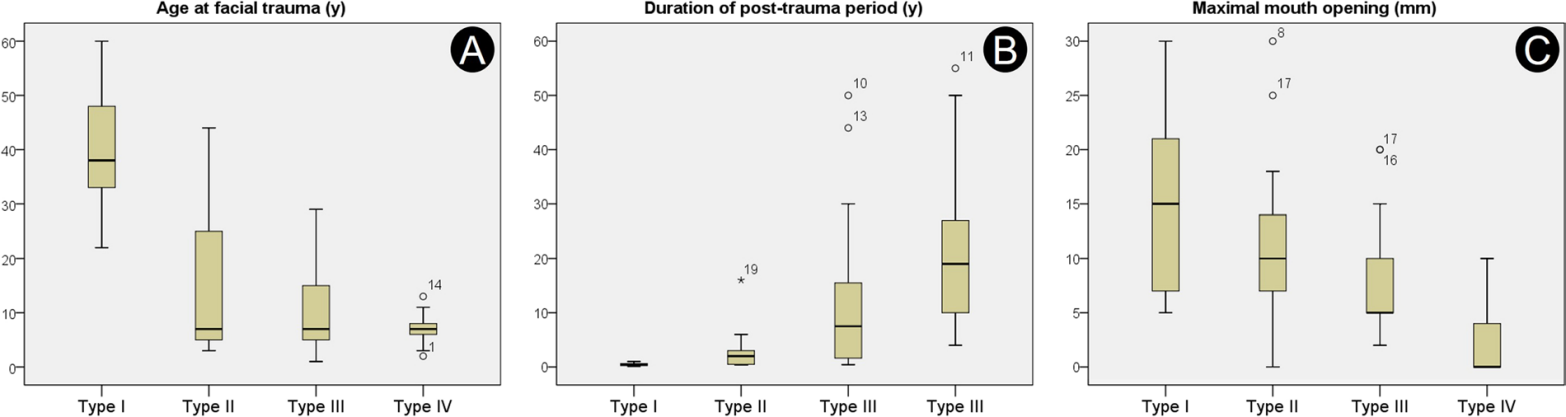
Box-plot diagram and correlation analysis by Spearman’s rank correlation coefficient. (**A**) Relationship between type and age at occurrence of maxillofacial trauma (r = −0.644; p < 0.05); (**B**) relationship between type of TMJ ankylosis and time after occurrence of maxillofacial trauma (r = 0.733; p < 0.05); (**C**) relationship between type of TMJ ankylosis and maximal mouth opening (r = −0.489; p < 0.05); TMJ, temporomandibular joint.

Correlation among patient age at occurrence of maxillofacial trauma, duration after maxillofacial trauma, and maximal mouth opening (MMO) were tested using Spearman’s rank correlation coefficient (Fig. 3). In the modified classification, MMO is highly negatively correlated with the severity of ankylosis (r = −0.644; p < 0.05). Younger trauma patients tended to experience more-severe types of ankylosis (r = −0.489; p < 0.05) than did older trauma patients. Additionally, long post-trauma periods before operation were related to the development of severe ankylosis (r = 0.733; p < 0.05).

### Intraoperative observations

**Figure 4:**
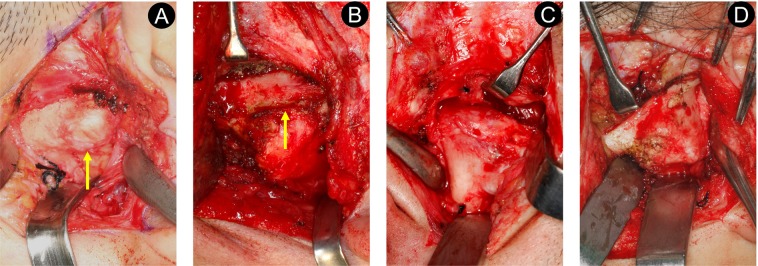
Intraoperative findings of ankylosed masses among the four types of temporomandibular joint ankylosis. Type I (**A**), non-bony; type II (**B**), lateral bony; type III (**C**), complete bony; type IV (**D**), extensive bony. (↑) represents the residual joint space. The residual joint space of type III and type IV was invisible to the naked eye.

In type I ankylosis, the surface of the residual condyle and glenoid fossa was rough. The disc was displaced in an anteromedial direction from the condyle, and the intact disc could be repositioned. The condyle could rotate but not slide. In type II ankylosis, a bony mass was formed around the lateral pole and lateral aspect of the fossa. After removing the bony mass, a pseudarthrosis formed between the medially displaced condyle and the fossa. The disc was generally intact, and reduction was possible by traction. Residual joint space and abnormal movement of the mandible were noted. In type III ankylosis, bone fusion was observed in the whole TMJ. The surface of the bony mass was substantially harder, and the residual joint space was invisible to the naked eye. There was almost no movement of the mandible. Reduction of the disc was difficult, even though the bony mass was completely cut. In type IV ankylosis, the whole TMJ developed into a large bony mass and its surface was similar to that of hard cortical bone. The disc was rarely seen (Fig. 4).

### Histopathological features

**Figure 5:**
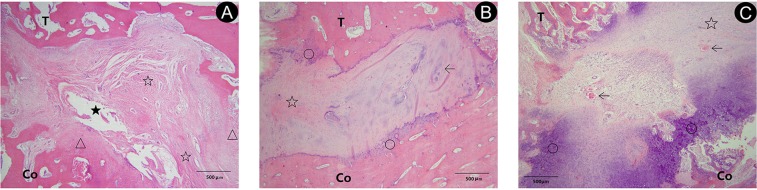
Histopathological features of type I (**A**), type II (**B**), and type III (**C**) temporomandibular joint ankylosis. Thickened cartilage cell layer (Δ); irregular fibrous tissue (☆); cracks and fissures of fibrous tissue (★); newly-formed capillaries (←); T: temporal bone; Co: condyle. Hematoxylin-eosin, 40×.

In type I ankylosis, the joint space was composed of irregular fibrous tissue. The cartilage cell layer on both sides was thickened, similar to the fibrocartilage tissue, and the middle of the joint space was filled with irregular fibrous connective tissue. Cracks and fissures were observed between fibrous tissues (Fig. 5). In type II ankylosis, the joint space was composed of layers of mostly fibrous and some cartilaginous tissue. The cartilage cell layer on both sides of the bone was composed of calcified cartilage close to the bone and uncalcified cartilage toward the joint space. One or more undulating hematoxyphil lines were present between the two layers of cartilage tissue. Fibrous tissue and capillary vessels were present in the residual joint space. The joint space in type III ankylosis was predominantly filled with cartilage, which was similar to the hyaline cartilage tissue. The chondrocytes were markedly hypertrophic and calcified, and extended toward the bone marrow cavities and joint space, suggesting that the osseous was active. In addition, fibrous connective tissue was observed and seemed to be involved vascular invasion. In type IV ankylosis, the joint space disappeared and was therefore not considered in the histological examination.

## Discussion

It is important to correctly estimate the severity of TMJ ankylosis in order to accurately evaluate the difficulty of surgery, precisely plan the resection, and assess the amounts of autogenous or alloplastic material required to fill the gap created in gap arthroplasty^11^. Extensive research has focused on determining the ideal algorithm for classification of the severity of TMJ ankylosis^12^. Our study proposed a modified classification system for TMJ ankylosis to allow provision of classification-based treatment suggestions for the condition. Using our classification system, all the patients in this study were grouped according to differences in the change in joint space and the radiolucent zone, and we found significant correlations and remarkable differences in the clinical features among the four groups.

In general, TMJ ankylosis can be classified by location (internal or external), the type of tissue involved (fibrotic or bony), and extent of ankylosis (partial or total)^5^. In 1986, Sawhney^13^ categorized TMJ ankylosis into four types according to radiography results. This method based on plain radiography has several advantages such as objective evaluation of scope of bony mass. However, based on our clinical experience, plain radiography has certain limitations in evaluating the condition of the residual condylar head and the degree of ossification, which thereby affects the prediction of probable surgical difficulties. The 2002 classification proposed by El-Hakim *et al*.^14^ was based on CT scans and focused on adjacent anatomic structures. In 2011, He *et al*.^15^ proposed a classification for evaluating the medially displaced condyle. Although previous systems have been useful for classification of TMJ ankylosis, these do not analyze sufficient data on manifestations of clinical features and disease development.

One of the classification standards—the joint space—coexists with a medially displaced condyle and constitutes the pseudarthrosis of the condyle-fossa^16^. Zheng *et al*.^17^ investigated the disc using magnetic resonance imaging and showed that the disc was discernible in all joints in lateral bony ankylosis, but with a degree of deformity and an intermediate position. Operative observation showed that the disc was generally intact and reduction was possible by traction. The optimal inter-positional material may have served as a barrier to prevent fusion of the condyle with the glenoid fossa^18–20^. Another classification standard—the radiolucent zone—is similar to the joint space, but differs due to its demonstration of insufficient calcification of the bony fusion area. Radiography showed that the radiolucent zone presented as a low-density line inside the fusion area. Its histopathological feature was the residual joint space manifested as a compound tissue structure of fibrous, cartilaginous, and osseous tissue. In our previous research^21^, CT findings were consistent with histologic results. Type II and III ankyloses were cartilaginous bony ankylosis, with similar components but with different degrees of severity. This could explain why some patients with bony ankylosis of TMJ can open their mouth to some degree but others cannot.

Ferretti *et al*.^22^ evaluated the joint morphology on coronal CT images and concluded that all the ankylosed joints exhibited a persistent rudimentary joint space. Ferretti *et al*.^22^ and Casanova *et al*.^23^ inferred that the radiolucent area inside the lesion represented a remnant of the inter-articular disc. We believe that the radiolucent zone may represent the course of bone healing of two traumatic articular surfaces with interference from the mouth-opening movements. This zone can be regarded as a mark of bony fusion development to a certain stage. Further research using magnetic resonance imaging is needed to verify this hypothesis.

In our study, when the radiolucent zone disappeared, the ankylosis progressed to the end stage (type IV in our classification). Both young age at occurrence of facial trauma (<10 years) and time after trauma occurrence (>10 years) were risk factors for the severe phase of ankylosis. Therefore, the growth and development of the maxillofacial region were inevitably affected, resulting in a high percentage of facial deformity. The treatment of type IV ankylosis should be directed toward the release of ankylosis and correction of the facial deformity. Furthermore, simultaneous or secondary mandibular advancement should be considered to enlarge the collapsed posterior airway space and relieve OSAS.

This study found a correlation of age at injury with prevalence and severity of TMJ ankylosis and concomitant complications, which is consistent with the finding of Kaur^24^. In addition, Anyanechi^25^ showed that patient age was correlated with TMJ ankylosis. Compared to mature adults, children had greater growth and reparative potential in the lamellar bony structures because they have rich vascularization. Allori *et al*.^4^ reported that the pediatric mandible is characterized by a broad condyle and thin cortical bone, which predispose children to intra-capsular comminuted fractures. In contrast, the elongated condylar neck in adults limits fractures to the extracapsular space. Bello *et al*.^1^ found that 17 of 23 patients with TMJ ankylosis had combined facial deformity, all of which had suffered maxillofacial trauma at <5 years of age.

In conclusion, this study highlights significant differences among the four types of traumatic TMJ ankylosis in terms of clinical features. Younger trauma patients with long post-trauma periods tend to develop more severe TMJ ankylosis, manifest lower MMO, experience more complications, and face more challenges in treatment than older patients. Our proposed modified classification system is efficient in evaluating the disease severity and developmental stage of traumatic TMJ ankylosis on the basis of CT findings. Moreover, all four types of ankylosis have precise definitions and distinctive criteria, which make it possible to achieve highly reproducible results and a high Kappa value.

Despite our important findings, this study has a few shortcomings. First, although the collection of imaging data on facial trauma would provide a deeper understanding and establish the relationship between the type of traumatic TMJ ankylosis and features of facial fracture, it was difficult to achieve. Second, MRI analysis was the best way to describe the joint disc of different type of ankylosis, but unfortunately, most of patients lacked of MRI examinations. In our further investigation, we will attempt to evaluate the articular disc based on MRI examinations. Thirdly, we were unable to conduct a follow-up for our patients, even though a follow-up is essential to further optimize our classification-based therapeutic strategy. Restricted by being a retrospective study, the conclusions of this study are less convincing than those of prospective studies. In further work, animal experiments could be used to test the conclusion whether younger trauma patients with long post-trauma periods tended to develop more severe TMJ ankylosis. Fourthly, TMJ ankylosis is an uncommon disease entity so this study is constrained by small numbers. A multicenter international work is needed to reconfirm the conclusions of this study.

## Methods

### Patients

This retrospective study included 71 patients with 102 ankylosed joints who visited the Department of Oral and Maxillofacial Surgery of the Peking University Hospital of Stomatology between January 2012 and December 2015. The study protocol was approved by the ethics committee of the Peking University School and Hospital of Stomatology [No. PKUSSIRB-201416095]. All study procedures were conducted in accordance with relevant guidelines and regulations. All participants provided written informed consent to publish identifying information/images and clinical records. Inclusion criterion were (i) time of visit between 2012 and 2015, (ii) TMJ ankylosis caused by trauma, and (iii) ankylosis confirmed by CT imaging. Exclusion criterion included (i) nontrauma factors, (ii) recurrent TMJ ankylosis, and (iii) lack of CT examination.

The study sample consisted of 42 men with 66 ankylosed joints and 29 women with 36 ankylosed joints. Of the 71 patients, 40 had unilateral ankylosis and 31 had bilateral ankylosis.

Basic information on age, gender, period after facial injury, MMO, concomitant facial deformity, and OSAS was obtained for all patients. CT examinations (helix with 1.25-mm slice thickness; Bright Speed 16, GE Healthcare, Buckinghamshire, UK) were performed, and multiplanar reformation was used to generate coronal CT images of the TMJ.

### Classification

Considering the change in joint space and the radiolucent zone on coronal CT, all ankylosed joints were categorized into one of four types (Fig. 1): type I, non-bony ankylosis of the joint with an almost-normal joint space and without bony fusion or a radiolucent line; type II, lateral bony ankylosis of the joint with lateral bony fusion and a radiolucent line inside the fusion area (medially discernible joint space coexisting with a displaced condyle); type III, complete bony ankylosis of the whole joint with a radiolucent zone inside the fusion mass but no clearly defined joint space; and type IV, extensive bony ankylosis with no clear definition of the joint or no radiolucent zone. When a patient with bilateral TMJ ankylosis had a different type of ankylosis on each side, the classification was based on the more critical side. All ankylosed joints were categorized by two surgeons who were members of the Department of Oral and Maxillofacial Surgery and were engaged in TMJ ankylosis research for >5 years. Any difference in opinion between the two surgeons was resolved by a third surgeon. The CT images were evaluated by three surgeons in a single-blind test.

### Surgery

During the surgery, the ankylosed mass was carefully exposed and harvested using osteotomy planes placed 3 mm above and 10 mm below the fusion area. The whole bony fusion mass was cut from the condyle to the temporal bone, with the residual joint space inside. Intraoperative findings such as movement of the remnant condyle, the characteristics of the residual joint space, and the condition of the articular disc were recorded.

### Histopathological examination

All specimens were fixed in 10% buffered formalin for 48 h and decalcified with 20% buffered ethylenediaminetetraacetic acid on a shaking table until elastic deformation occurred. After dehydration in a series of graded alcohols, the specimens were paraffin-embedded, cut into 4-μm serial sections, and stained with hematoxylin and eosin.

### Statistical analysis

The coverage rate was calculated, and the reliability of the test was determined by the coefficient of internal consistency using the Kappa value. The Spearman correlation coefficient was used to evaluate the relationship between the severity of ankylosis and clinical features, including age at occurrence of maxillofacial trauma, duration after facial trauma, and MMO. The χ^2^ and Fisher’s exact tests were used for intergroup comparisons of the proportion of facial deformity and OSAS among the different types of ankylosis. Statistical analysis was conducted using SPSS 19.0 software (SPSS Inc., Chicago, IL, USA). The level of statistical significance was set at p < 0.05.

### Ethical approval

This study was approved by the ethics committee of Peking University School and Hospital of Stomatology [No. PKUSSIRB-201416095]. All study procedures were conducted in accordance with relevant guidelines and regulations. All participants provided written informed consent to publish identifying information/images and clinical records.

## Data Availability

All data included in this study are available upon request by contact with the corresponding author.
